# Two-stage strategy using denoising autoencoders for robust reference-free genotype imputation with missing input genotypes

**DOI:** 10.1038/s10038-024-01261-6

**Published:** 2024-06-25

**Authors:** Kaname Kojima, Shu Tadaka, Yasunobu Okamura, Kengo Kinoshita

**Affiliations:** 1 Tohoku Medical Megabank Organization, Tohoku University, 2-1 Seiryo-machi, Aoba-ku, Sendai, Miyagi 980-8573 Japan; 2 Advanced Research Center for Innovations in Next-Generation Medicine, Tohoku University, 2-1 Seiryo-machi, Aoba-ku, Sendai, Miyagi 980-0873 Japan; 3 Graduate School of Information Sciences, Tohoku University, 6-3-09 Aza-Aoba, Aramaki, Aoba-ku, Sendai, Miyagi 980-8579 Japan; 4 Institute of Development, Aging and Cancer, Tohoku University, 4-1 Seiryo-machi, Aoba-ku, Sendai, Miyagi 980-8575 Japan

**Keywords:** Genetics, Biomarkers

## Abstract

Widely used genotype imputation methods are based on the Li and Stephens model, which assumes that new haplotypes can be represented by modifying existing haplotypes in a reference panel through mutations and recombinations. These methods use genotypes from SNP arrays as inputs to estimate haplotypes that align with the input genotypes by analyzing recombination patterns within a reference panel, and then infer unobserved variants. While these methods require reference panels in an identifiable form, their public use is limited due to privacy and consent concerns. One strategy to overcome these limitations is to use de-identified haplotype information, such as summary statistics or model parameters. Advances in deep learning (DL) offer the potential to develop imputation methods that use haplotype information in a reference-free manner by handling it as model parameters, while maintaining comparable imputation accuracy to methods based on the Li and Stephens model. Here, we provide a brief introduction to DL-based reference-free genotype imputation methods, including RNN-IMP, developed by our research group. We then evaluate the performance of RNN-IMP against widely-used Li and Stephens model-based imputation methods in terms of accuracy (R^2^), using the 1000 Genomes Project Phase 3 dataset and corresponding simulated Omni2.5 SNP genotype data. Although RNN-IMP is sensitive to missing values in input genotypes, we propose a two-stage imputation strategy: missing genotypes are first imputed using denoising autoencoders; RNN-IMP then processes these imputed genotypes. This approach restores the imputation accuracy that is degraded by missing values, enhancing the practical use of RNN-IMP.

## Introduction

High-throughput sequencing technologies have enabled the construction of genotype datasets at base-level resolution for thousands of individuals. These datasets are phased to form collections of haplotypes, known as haplotype reference panels. Genotype imputation, one of the key applications of reference panels, leverages them to estimate the genotypes at unobserved segregating sites with sequence-level resolution. While SNP (Single Nucleotide Polymorphism) array technology enables cost-effective genotyping, the obtained genotypes are restricted to pre-designed markers. Genotype imputation resolves this limitation by estimating genotypes at unobserved segregating sites, thereby enhancing the detection of trait-associated variants in genome-wide association studies (GWAS), and improving the accuracy of trait heritability and polygenic risk score calculations [1–3].

The widely used genotype imputation methods, such as IMPUTE [4–7], Minimac [8], and Beagle [9], are based on the Li and Stephens model [10]. This model assumes that new haplotypes can be represented by modifying existing ones in the reference panel through a series of mutations and recombinations. These methods use phased genotypes from SNP arrays as inputs and estimate haplotypes that align with these inputs by analyzing how haplotypes in the reference panel recombine. Subsequently, the genotypes of unobserved variants can be inferred from these estimated haplotypes. While imputation methods based on the Li and Stephens model require a haplotype reference panel in its raw, identifiable form, the availability of such detailed haplotype data is generally limited due to privacy concerns and the need for donor consent for public use. For instance, the Trans-Omics for Precision Medicine (TOPMed) program offers an imputation server service using its large haplotype reference panel [11], based on the Michigan Imputation Server system [8]. The haplotype reference panel used in the system is comprised of the genotype data of 133,597 individuals across various populations, but the access to the haplotype reference panel itself is not publicly available. Similarly, the Northeast Asian Reference Database (NARD) provides a haplotype reference panel comprised of 1,779 Northeast Asian individuals, but its access is also limited to use through its imputation server system [12]. Consequently, to use non-public haplotypes for more accurate imputation, researchers must transmit input genotype data to research institutes that maintain their own private haplotype databases, although the sharing of this input genotype data can be further complicated by informed consent agreements that often restrict external use.

One strategy to address these restrictions is to use haplotype information in a de-identified manner, such as through summary statistics or model parameters, from which the reconstruction of genotype data at the individual level is nearly impossible. For example, combining GWAS summary statistics allows the calculation of p-values across cohorts without sharing individual-level genotypes [13]. Methods addressing sample overlap issues in the combination of GWAS summary statistics have also been proposed [14–16]. In HLA type imputation, HIBAG employs attribute-bagging to infer HLA types from SNP array genotypes, and once the model parameters are trained, HLA types can be estimated in a reference-free manner [17]. Choudhury et al. proposed a reference-free imputation method using support vector machines (SVMs), in which the SVM parameters are trained with a reference panel, and genotypes at variant sites in the reference panel are estimated from trained SVMs [18]. Although this SVM-based method is less demanding in terms of computation and memory, its accuracy is not as high as that of methods based on the Li and Stephens model.

Recent advancements in deep learning (DL) have demonstrated significant enhancements across various fields and offer the potential to develop reference-free imputation methods that can process haplotype information in a de-identified manner—namely, as model parameters—while maintaining imputation accuracy comparable to that of methods based on the Li and Stephens model. Chen and Shi proposed a reference-free imputation method using sparse convolutional denoising autoencoders (SCDA) for imputing missing genotypes with a relatively low missing rate, such as less than 0.2 [19]. Their model incorporates convolutional layers with L1 regularization to encourage sparsity in kernel values. The model is trained by fitting randomly masked input genotypes to the original genotypes. This method has demonstrated higher accuracy compared to traditional methods like KNN or singular value decomposition. The number of target imputation sites significantly exceeds the number of input genotype positions in a scenario commonly addressed by widely-used Li and Stephens model-based methods. For such a scenario, our research group proposed a reference-free imputation method named RNN-IMP [20, 21]. RNN-IMP employs a bidirectional recurrent neural network (RNN) and has provided enhanced imputation accuracy in comparison to the SVM-based method by Choudhury et al., and the accuracy is competitive with the widely-used Li and Stephens model-based methods. Other DL-based genotype imputation approaches include the work of Dias et al., who used a sparse denoising autoencoder (DAE) in a similar scenario to RNN-IMP [22], while Dias’ method employed fully connected layers, as opposed to the convolutional approach of Chen and Shi. Dias’ method was less accurate compared to those of methods based on the Li and Stephens model in the initial setting, but additional fine-tuning using simulated offspring data and optimized hyperparameters resulted in improved imputation accuracy over the Li and Stephens model-based methods. Song et al. refined the SCDA training process by employing single batch loss instead of average loss over multiple batches to update parameters [23]. Naito et al. developed a CNN-based method named DEEP*HLA for estimating HLA types from SNP array genotypes [24]. Mowlaei et al. introduced a transformer-based genotype imputation method named Split-Transformer Impute (STI), which primarily focuses on structural variations and has demonstrated greater imputation accuracy for these variations than existing methods, including those based on the Li and Stephens model [25].

Although DAE-based methods like SCDA are robust to missing input values—as these are incorporated into the training process — methods such as RNN-IMP and other supervised learning-based approaches that do not account for missing input values during training can be vulnerable to them. In SNP array data analysis, specific markers may be removed during quality control, for instance, due to deviations from Hardy-Weinberg equilibrium or low call rates. This susceptibility to missing genotypes is a significant disadvantage for practical applications. Since DAE-based methods are robust to missing input values and are also suitable for imputing those values, we propose a two-stage genotype imputation strategy. Initially, this strategy employs DAE-based methods like SCDA to impute missing values in either phased or unphased genotypes from SNP arrays. In the second step, these imputed genotypes are used as input for genotype imputation methods such as RNN-IMP, to estimate genotypes at variant sites in reference panels.

In the sections that follow, we first verify the imputation accuracy of RNN-IMP by comparing it with widely-used Li and Stephens model-based methods, using phased genotypes with no missing values as input. We also compare the computational time required by these methods. We then assess the impact of missing values in the input genotypes on imputation accuracy and evaluate the effectiveness of our two-stage genotype imputation strategy against the missing values.

## Results

### Train and test data preparation

We used phased genotype data from chromosome 22 of 2504 individuals in the phase 3 dataset of the 1000 Genomes Project (1KGP) [26] (https://ftp.1000genomes.ebi.ac.uk/vol1/ftp/release/20130502/ALL.chr22.phase3_shapeit2_mvncall_integrated_v5b.20130502.genotypes.vcf.gz) to prepare the evaluation dataset. For testing, we randomly selected 100 individuals and assessed the imputation performance using the phased genotype data of the remaining 2,404 individuals as the haplotype reference panel. These 100 individuals are listed in Supplementary Table 1. We employed the Infinium Omni2.5-8 BeadChip (Omni2.5-8 v1.4 kit version), hereafter referred to as Omni2.5, as the SNP array platform and extracted genotypes at 31,325 sites designed on Omni2.5 as input data for genotype imputation methods. We evaluated the imputation accuracy for genotypes at the remaining 1,078,043 sites. Note that we directly used phasing information from the reference panel for the input genotypes; therefore, no prephasing process was applied in this experiment. Since RNN-IMP requires substantial computational resources for model training, accommodating all 1,078,043 sites would be computationally intensive, we filtered out rare variants with a minor allele frequency (MAF) of less than 0.005 as the target sites for RNN-IMP. After this filtering, the number of target sites was reduced to 217,428.

### Evaluation of imputation accuracy in R^2^ for input genotypes without missing values

We compared the genotype imputation accuracy of RNN-IMP with methods based on the Li and Stephens model, as listed in Table 1, using test input genotypes without missing values.

**Table 1:** List of genotype imputation methods based on the Li and Stephens model evaluated in this study

Genotype imputation method	Version
IMPUTE5	v1.2.0
IMPUTE4	v4.1.2_r300.3
Beagle 5.4	version 22Jul22.46e
Minimac4	v4.1.6
Minimac3	v2.0.1

For the RNN-IMP model configuration, we set the number of layers and the size of the hidden vectors to 4 and 40, respectively. We divided chromosome 22 into linkage disequilibrium (LD) blocks using the LD block information in https://github.com/stephenslab/ldshrink/blob/main/inst/test_gdsf/fourier_ls-all.bed. Each block was further divided to ensure that each resulting region contained approximately 200 array markers, resulting in a total of 225 regions. Variant sites located within each subdivided region, except array markers, were designated as outputs for the corresponding RNN model. 50 array markers on both the upstream and downstream sides of each region were also included for the model inputs. We employed a similar approach to that described in [21] for training the model parameters.

**Fig. 1:**
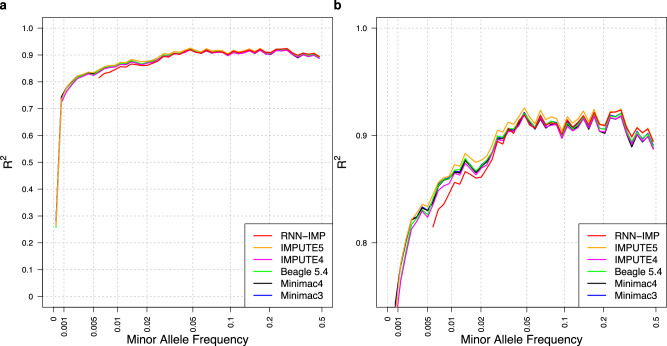
**a** A plot comparing R^2^ values of genotype imputation methods. **b** A plot with a y-axis range limited to R^2^ ≥ 0.7

Default options were used for the Li and Stephens model-based methods. Within the evaluation dataset, all variant sites were biallelic; one allele was denoted as the a0 allele, and the other as the a1 allele. To assess genotype imputation accuracy, we used the R^2^ value, calculated as the square of the Pearson correlation coefficient between the actual genotype counts of the a1 allele and the a1 allele dosages in the imputed genotypes. Figure 1a presents a comparison of R^2^ values for RNN-IMP and the methods listed in Table 1 across the MAF spectrum. The R^2^ values on the y-axis represent the average R^2^ values for variants within small local bins for each MAF value. Figure 1b displays the comparison of the R^2^ values with a y-axis range limited to values ≥0.7 to focus on this range. Although the differences among the Li and Stephens model-based methods are slight, IMPUTE5 exhibits the highest R^2^ values across most of the MAF range, except for very rare variants (MAF < 0.001), where Minimac3 presents the highest. From the comparison in R^2^, RNN-IMP demonstrates competitive accuracy relative to the Li and Stephens model-based methods for input genotypes without missing values.

### Comparison of computational time

We recorded the computational time required for obtaining genotype imputation results with RNN-IMP and the Li and Stephens model-based methods, as summarized in Table 2. Since model training was required for RNN-IMP, the running time on this process was also recorded. IMPUTE5, Minimac3, and Minimac4 require a preprocessing phase to reformat the reference panel data for efficient computation, and the duration of this step was also recorded.

**Table 2:** Summary of the computational time required for genotype imputation including preprocessing and model training time where applicable for each method

Method	Genotype imputation time	Preprocessing or training time
IMPUTE5	70.35s	11.57s
IMPUTE4	162.13s	−
Beagle5.4	24.97s	−
Minimac4	54.64s	20110.55s (5h 35m 10.55s, 12.46s for converting m3vcf to msav)
Minimac3	1534.54s	20098.09s (5h 34m 58.09s)
RNN-IMP	584.30s	30440256.56s (352d 7h 37m 36.56s)

Minimac4 requires the reference panel data to be in msav format. While this format can be directly obtained from vcf format reference panel data, it can also be derived from m3vcf format, which is used by the previous version, Minimac3. Although the direct conversion from vcf to msav takes significantly less time than the conversion from vcf to msav via m3vcf, we observed that the imputation accuracy using msav format data obtained through the direct process was lower than that achieved with data converted via m3vcf. Based on our observations, we here opted to use msav data converted from m3vcf for our evaluation dataset, although this difference in accuracy is expected to diminish for large biobank-scale reference panels. We included the conversion time of m3vcf using Minimac3 in the preprocessing time for Minimac4.

Similar preprocessing, such as the data conversion with the Positional Burrows Wheeler Transform, is required for IMPUTE4 and Beagle 5.4; however, since these steps are integrated within the main genotype imputation process, their computational times were not recorded separately. The trained models or preprocessed data can be reused, and hence in Table 2, we distinguished this initial computational time from the subsequent running time for genotype imputation. The training time for RNN-IMP was assessed on an AMD Epyc 7713 CPU (base clock 2.0 GHz; boost clock up to 3.675 GHz) in a single thread, while other processes were measured on an AMD Ryzen 9 7950X CPU (base clock 4.5 GHz; boost clock up to 5.7 GHz) in a single thread. Python 3.7 and TensorFlow 1.15.0 were used for training the deep learning models for RNN-IMP. The trained models were then converted to the ONNX Runtime format (https://onnxruntime.ai/) to accelerate computations during model inference for genotype imputation. For inference, we used Python 3.11 with the ONNX Runtime. Although RNN-IMP demands a substantial amount of computational time for training, this process can be parallelized, and the entire training was completed within two days using a supercomputing system. RNN-IMP also generally consumed more computational time than methods based on the Li and Stephens model. One reason is that genotype data reading and result writing operations are implemented in Python for RNN-IMP, whereas they are implemented in C, C++, or Java in the Li and Stephens model-based methods. The inference phase by ONNX Runtime took 296.62 seconds for RNN-IMP, suggesting that computational efficiency could be improved by implementing I/O processes in C or C++ rather than Python. In this experiment, only 100 samples were processed for genotype imputation, but it is anticipated that the computational time per sample will decrease significantly for larger datasets, due to reduced overhead in all methods.

### Evaluation of imputation accuracy in R^2^ for input genotypes with missing values

**Fig. 2:**
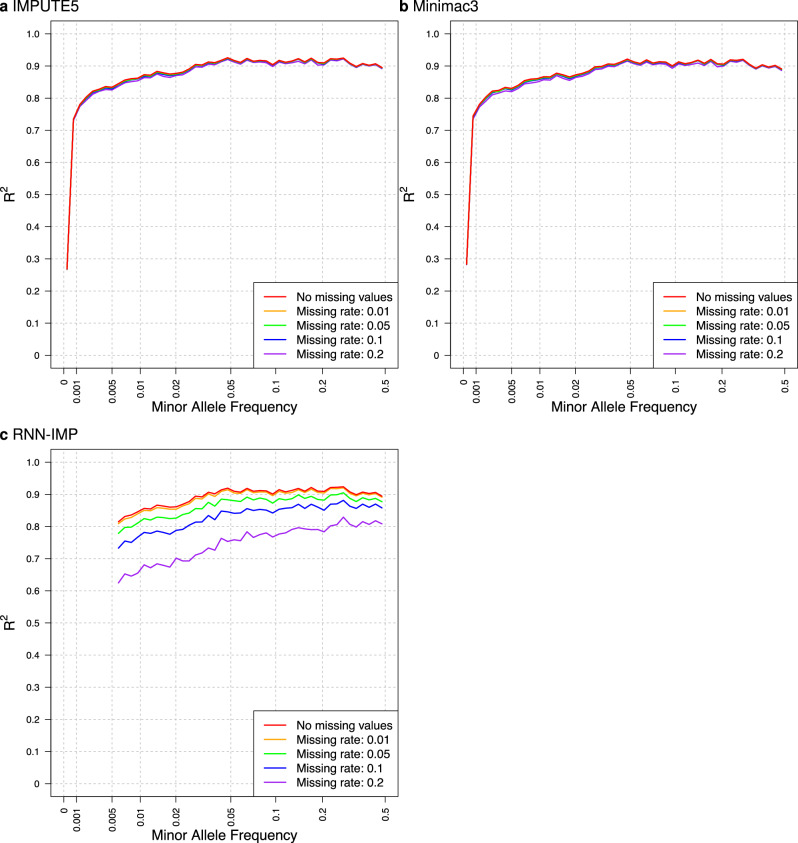
Comparison of R^2^ values for input genotypes without missing values and for those with missing values at rates of 0.01, 0.05, 0.1, and 0.2, across each imputation method: **a** IMPUTE5, **b** Minimac3, and **c** RNN-IMP

In SNP array genotype analysis, certain markers are removed during quality control, for instance, due to deviations from the Hardy-Weinberg equilibrium or low call rates. Since methods based on the Li and Stephens model use hidden Markov models or state space models, missing values in the input genotypes are simply skipped, and there is no need to fill in alternative values for the removed markers. On the other hand, RNN-IMP treats input genotypes as model inputs and does not account for missing values in input genotypes during the training process. Thus, some value must be supplied for the removed markers. The input value of RNN-IMP for each marker is represented by a one-hot vectors indicating the presence of a0 and a1 alleles, and the vector [1 − *f*_a1_, *f*_a1_], where *f*_a1_ denotes the frequency of the a1 allele, is used for missing values. To assess the impact of missing values in input genotypes on each method, we simulated the removal of genotype data for some markers in the test input data. Since the rate of removal due to quality control is typically at most 0.2, we considered the following removal rates: 0.01, 0.05, 0.1, and 0.2. Figure 2a, b, and c illustrate the impact of missing inputs on the performance of IMPUTE5, Minimac3, and RNN-IMP, respectively. In this comparison, we selected IMPUTE5 and Minimac3 as representative methods of those based on the Li and Stephens model and omitted other methods from this evaluation. As expected, the impact of missing input genotypes was negligible for the Li and Stephens model-based methods, while a clear reduction in R^2^ values was observed for RNN-IMP as missing rates increased.

### Two-stage strategy for robust genotype imputation to missing values

**Fig. 3:**
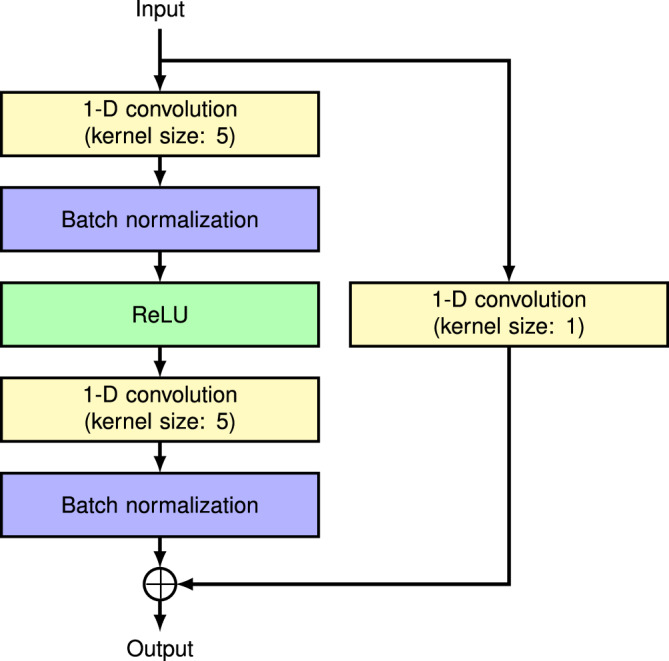
The structure of residual 1D-convolution block in RCDA

In order to impute missing values in the input genotypes, we considered a modified version of the DAE model used in [19]. Table 3a shows the original DAE model architecture, which uses a kernel size of 5 in its 1-D convolution layers and incorporates L1 regularization to encourage sparsity in the kernel parameters. In our modification, we replaced the 1D-convolution layers with residual 1D-convolution blocks as illustrated in Fig. 3. Residual connections, also known as skip connections, are the key feature of residual blocks and are effective for the efficient training of deep learning models by preventing the vanishing and exploding gradients during the backpropagation process [27]. We anticipated that the use of residual blocks would enhance the performance in the DAE-based methods. Since batch normalization within residual blocks helps prevent overfitting, we also omitted the dropout layers and L1 regularization previously applied to the 1D-convolution kernels. The output vector for each variant site in the original model was a one-hot vector representing three states: missing, a0 allele, and a1 allele. Since the missing state in output is not required for our purpose, we modified the output vector to only represent the a0 and a1 alleles. We have named this residual convolution denoising autoencoder-based method as RCDA. Table 3b shows the model architecture of RCDA. Similar to the approach used for RNN-IMP, chromosome 22 was divided into LD blocks. Each region was then further segmented to contain approximately 500 array markers. In total, 75 regions were obtained. For training RCDA, we employed the Adam optimizer [28] configured for 1,000 epochs, a learning rate of 0.001, and a batch size of 32. We selected the parameters that produced the best loss for a randomly selected set of 100 validation samples as the trained parameters for the model for each region. Table 4 summarizes the computational times for genotype imputation and model training using RCDA. Training time as well as genotype imputation time were evaluated on an AMD Ryzen 9 7950X CPU in a single thread. We also evaluated the training time using GPU computation with a GeForce RTX 4090 along with an AMD Ryzen 9 7950X CPU in a single thread. Training with the GPU was approximately three times faster than using only the CPU in a single thread. However, since models for different regions can be trained independently, parallel training across multiple CPU cores is a promising option for accelerating computation. Due to the overhead associated with GPU computation, using the GPU for this dataset resulted in greater overall computational time compared to using only the CPU in the inference for genotype imputation. Genotype imputation time also can be reduced by using multiple CPU cores to independently process models for different regions.

**Table 3:** The model architectures of **a** DAE used in [19] and **b** RCDA

Layer Position	Layer Type	Output Channel Size
**a**		
1st layer	1-D convolution	32
2nd layer	ReLU	32
3rd layer	Max pooling (pool size: 2)	32
4th layer	Dropout	32
5th layer	1-D convolution	64
6th layer	ReLU	64
7th layer	Max pooling (pool size: 2)	64
8th layer	Dropout	64
9th layer	1-D convolution	128
10th layer	ReLU	128
11th layer	1-D convolution	64
12th layer	ReLU	64
13th layer	Upsampling (size: 2)	64
14th layer	Dropout	64
15th layer	1-D convolution	32
16th layer	ReLU	32
17th layer	Upsampling (size: 2)	32
18th layer	Dropout	32
19th layer	1-D convolution	3
20th layer	Softmax	3

b		
1st layer	Residual 1D-convolution block	32
2nd layer	ReLU	32
3rd layer	Max pooling (pool size: 2)	32
4th layer	Residual 1D-convolution block	64
5th layer	ReLU	64
6th layer	Max pooling (pool size: 2)	64
7th layer	Residual 1D-convolution block	128
8th layer	ReLU	128
9th layer	Residual 1D-convolution block	64
10th layer	ReLU	64
11th layer	Upsampling (size: 2)	64
12th layer	Residual 1D-convolution block	32
13th layer	ReLU	32
14th layer	Upsampling (size: 2)	32
15th layer	1-D convolution	2
16th layer	Softmax	2

**Table 4:** Computational time for genotype imputation and model training in RCDA

Missing rate	Genotype imputation time	Training time using GPU	Training time using CPU only
0.01	27.06s	104445.06s (1d 5h, 45.06s)	292144.95s (3d 9h, 9m, 4.95s)
0.05	27.14s		
0.1	27.19s		
0.2	27.16s		

**Fig. 4:**
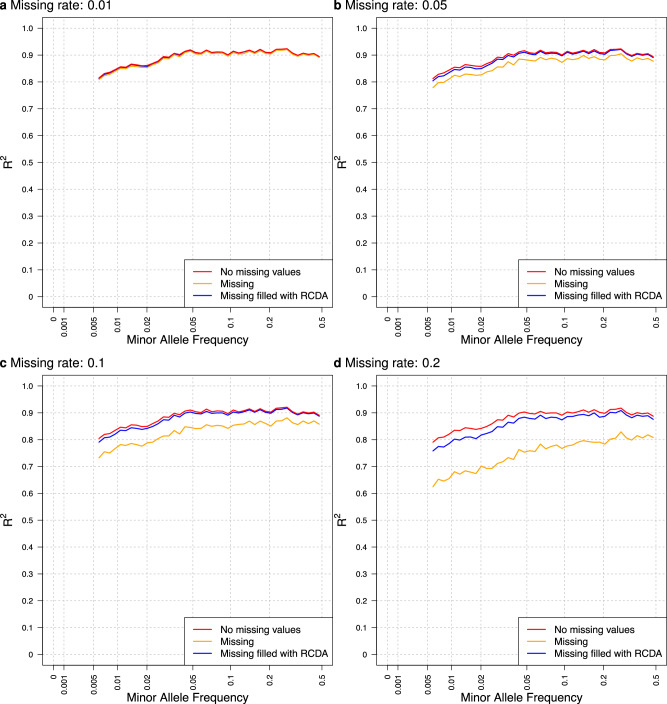
R^2^ values for RNN-IMP for input genotypes without missing values, those with missing values, and those with missing values imputed with RCDA, across missing rates of **a** 0.01, **b** 0.05, **c** 0.1, and **d** 0.2

Figure 4 shows a comparison of R^2^ values for genotype imputations among original input genotypes without missing values, those with missing values, and those with missing values imputed by RCDA across missing rates of 0.01, 0.05, 0.1, and 0.5. The R^2^ values demonstrate substantial recovery following the imputation of missing genotypes by RCDA, while this recovery is less pronounced for variants with lower MAF, which may be attributed to the reduced imputation accuracy of RCDA for such markers in SNP arrays.

## Conclusion

We confirmed the competitive imputation accuracy (R^2^) of RNN-IMP compared to widely-used imputation methods based on the Li and Stephens model, using the 1KGP Phase 3 phased genotype dataset and simulated Omni2.5 SNP array genotype data derived from the dataset. In terms of running time, RNN-IMP generally required more time than the Li and Stephens model-based methods. However, the running time for RNN-IMP is not dependent on the size of the reference panel, suggesting that the time difference could diminish with larger reference panels. Although the total time for training RNN models was substantial, the models can be trained concurrently, thus allowing the training process to be completed within a reasonable number of days using massively parallel computing resources. Unlike the methods based on the Li and Stephens model, the presence of missing values in the input genotypes significantly reduced imputation accuracy for RNN-IMP. To address this issue, we introduced a two-stage genotype imputation strategy where missing genotypes are first imputed using DAE, and the resulting imputed genotypes are then used as inputs for RNN-IMP. This strategy substantially restored imputation accuracy in terms of R^2^, enhancing the practical application of RNN-IMP. While the efficacy of the two-stage strategy was demonstrated only for RNN-IMP, this framework is broadly applicable. In future work, we plan to assess the utility of the two-stage strategy for other supervised learning-based genotype imputation methods that do not account for missing values in the training process, such as HIBAG and DEEP*HLA.

## Use of large language models

We used ChatGPT (https://chat.openai.com) only for improving the readability of texts.

## Supplementary information

Supplemental table1

## Data Availability

The code for RNN-IMP and RCDA, along with the model parameters obtained from the training data used in this study, is publicly available on the following GitHub pages: • RNN-IMP: https://github.com/kanamekojima/rnnimp • RCDA: https://github.com/kanamekojima/RCDA Instructions for generating the evaluation datasets used in this study are also provided in the README files on these GitHub pages.

## References

[1] Marchini J, Howie B. Genotype imputation for genome-wide association studies. Nat Rev Genet. 2010;11:499–511. 20517342 10.1038/nrg2796

[2] Duncan L, Shen H, Gelaye B, Meijsen J, Ressler K, Feldman M, et al. Analysis of polygenic risk score usage and performance in diverse human populations. Nat Commun. 2019;10:3328. 31346163 10.1038/s41467-019-11112-0 PMC6658471

[3] Yang J, Bakshi A, Zhu Z, Hemani G, Vinkhuyzen AA, Lee SH, et al. Genetic variance estimation with imputed variants finds negligible missing heritability for human height and body mass index. Nat Genet. 2015;47:1114–20. 26323059 10.1038/ng.3390 PMC4589513

[4] Howie BN, Donnelly P, Marchini J. A flexible and accurate genotype imputation method for the next generation of genome-wide association studies. PLoS Genet. 2009;5:e100052. 10.1371/journal.pgen.1000529 PMC2689936 19543373

[5] Howie B, Marchini J, Stephens M. Genotype imputation with thousands of genomes. G3: Genes, Genomes, Genet. 2011;1:457–70. 10.1534/g3.111.001198 PMC3276165 22384356

[6] Bycroft C, Freeman C, Petkova D, Band G, Elliott LT, Sharp K, et al. The UK Biobank resource with deep phenotyping and genomic data. Nature. 2018;562:203–9. 30305743 10.1038/s41586-018-0579-z PMC6786975

[7] Rubinacci S, Delaneau O, Marchini J. Genotype imputation using the Positional Burrows Wheeler Transform. PLoS Genet. 2020;16:e1009049. 33196638 10.1371/journal.pgen.1009049 PMC7704051

[8] Das S, Forer L, Schönherr S, Sidore C, Locke AE, Kwong A, et al. Next-generation genotype imputation service and methods. Nat Genet. 2016;48:1284–87. 27571263 10.1038/ng.3656 PMC5157836

[9] Browning BL, Zhou Y, Browning SR. A one-penny imputed genome from next generation reference panels. Am J Hum Genet. 2018;103:338–48. 30100085 10.1016/j.ajhg.2018.07.015 PMC6128308

[10] Li N, Stephens M. Modelling linkage disequilibrium and identifying recombination hotspots using single-nucleotide polymorphism data. Genetics. 2003;165:2213–33. 14704198 10.1093/genetics/165.4.2213 PMC1462870

[11] Taliun D, Harris DN, Kessler MD, Carlson J, Szpiech ZA, Torres R, et al. Sequencing of 53,831 diverse genomes from the NHLBI TOPMed Program. Nature. 2021;590:290–9. 33568819 10.1038/s41586-021-03205-y PMC7875770

[12] Yoo SK, Kim CU, Kim HL, Kim S, Shin JY, Kim N, et al. NARD: whole-genome reference panel of 1779 Northeast Asians improves imputation accuracy of rare and low-frequency variants. Genome Med. 2019;11:64. 31640730 10.1186/s13073-019-0677-z PMC6805399

[13] Niu YF, Ye C, He J, Han F, Guo LB, Zheng HF, et al. Reproduction and in-depth evaluation of genome-wide association studies and genome-wide meta-analyses using summary statistics. G3: Genes, Genomes, Genet. 2017;7:943–52. 10.1534/g3.116.038877 PMC5345724 28122950

[14] Lin DY, Sullivan PF. Meta-analysis of genome-wide association Studies with overlapping subjects. Am J Hum Genet. 2009;85:862–72. 20004761 10.1016/j.ajhg.2009.11.001 PMC2790578

[15] Chen GB, Lee SH, Robinson MR, Trzaskowski M, Zhu ZX, Winkler TW, et al. Across-cohort QC analyses of GWAS summary statistics from complex traits. Eur J Hum Genet. 2017;25:137–46. 10.1038/ejhg.2016.106 PMC5159754 27552965

[16] LeBlanc M, Zuber V, Thompson WK, Andreassen OA, Schizophrenia and Bipolar Disorder Working Groups of the Psychiatric Genomics Consortium, Frigessi A, et al. A correction for sample overlap in genome-wide association studies in a polygenic pleiotropy-informed framework. BMC Genomics. 2018;19:494. 29940862 10.1186/s12864-018-4859-7 PMC6019513

[17] Zheng X, Shen J, Cox C, Wakefield JC, Ehm MG, Nelson MR, et al. HIBAG-HLA genotype imputation with attribute bagging. Pharmacogenomics J. 2014;14:192–200. 23712092 10.1038/tpj.2013.18 PMC3772955

[18] Choudhury O, Chakrabarty A, Emrich SJ. Highly accurate and efficient data-driven methods for genotype imputation. IEEE/ACM Trans Comput Biol Bioinforma. 2019;16:1107–16. 10.1109/TCBB.2017.2708701 28574365

[19] Chen J, Shi X. Sparse convolutional denoising autoencoders for genotype imputation. Genes. 2019;10:652. 31466333 10.3390/genes10090652 PMC6769581

[20] Kojima K, Tadaka S, Katsuoka F, Tamiya G, Yamamoto M, Kinoshita K. A recurrent neural network based method for genotype imputation on phase genotype data, bioRxiv. 2019. 10.1101/821504v1. 10.1371/journal.pcbi.1008207 PMC7529210 33001993

[21] Kojima K, Tadaka S, Katsuoka F, Tamiya G, Yamamoto M, Kinoshita K. A genotype imputation method for de-identified haplotype reference information by using recurrent neural network. PLoS Comput Biol. 2020;16:e1008207. 33001993 10.1371/journal.pcbi.1008207 PMC7529210

[22] Dias R, Evans D, Chen S, Chen K, Loguercio S, Chan L, et al. Rapid, reference-free human genotype imputation with denoising autoencoders. eLife. 2022;11:e75600. 36148981 10.7554/eLife.75600 PMC9555874

[23] Song M, Greenbaum J, Luttrell IVth J, Zhou W, Wu C, Luo Z, et al. An autoencoder-based deep learning method for genotype imputation. Front Artif Intell. 2022;5:1028978. 36406474 10.3389/frai.2022.1028978 PMC9671213

[24] Naito T, Suzuki K, Hirata J, Kamatani Y, Matsuda K, Toda T, et al. A deep learning method for HLA imputation and trans-ethnic MHC fine-mapping of type 1 diabetes. Nat Commun. 2021;12:1639. 33712626 10.1038/s41467-021-21975-x PMC7955122

[25] Mowlaei ME, Li C, Chen J, Jamialahmadi B, Kumar S, Rebbeck TR, et al. Split-transformer impute (STI): genotype imputation using a transformer-based model, bioRxiv. 2023. 10.1101/2023.03.05.531190v1.

[26] 1000 Genomes Project Consortium, Auton A, Brooks LD, Durbin RM, Garrison EP, Kang HM, et al. A global reference for human genetic variation. Nature. 2015;526:68–74. 26432245 10.1038/nature15393 PMC4750478

[27] He K, Zhang X, Ren S, Sun J. Deep residual learning for image recognition, *Proceedings of the IEEE Conference on Computer Vision and Pattern Recognition* 2016, 770–78.

[28] Kingma D, Ba J. Adam: A method for stochastic optimization, *The 3rd International Conference on Learning Representations* (2015).

